# Biomechanics of *Tetrahymena* escaping from a dead end

**DOI:** 10.1098/rspb.2017.2368

**Published:** 2018-02-28

**Authors:** Takuji Ishikawa, Kenji Kikuchi

**Affiliations:** Department of Finemechanics, Graduate School of Engineering, Tohoku University, 6-6-01 Aoba, Aramaki, Aoba-ku, Sendai 980-8579, Japan

**Keywords:** ciliate, swimming, fluid mechanics, flagella

## Abstract

Understanding the behaviours of swimming microorganisms in various environments is important for understanding cell distribution and growth in nature and industry. However, cell behaviour in complex geometries is largely unknown. In this study, we used *Tetrahymena thermophila* as a model microorganism and experimentally investigated cell behaviour between two flat plates with a small angle. In this configuration, the geometry provided a ‘dead end' line where the two flat plates made contact. The results showed that cells tended to escape from the dead end line more by hydrodynamics than by a biological reaction. In the case of hydrodynamic escape, the cell trajectories were symmetric as they swam to and from the dead end line. Near the dead end line, *T. thermophila* cells were compressed between the two flat plates while cilia kept beating with reduced frequency; those cells again showed symmetric trajectories, although the swimming velocity decreased. These behaviours were well reproduced by our computational model based on biomechanics. The mechanism of hydrodynamic escape can be understood in terms of the torque balance induced by lubrication flow. We therefore conclude that a cell's escape from the dead end was assisted by hydrodynamics. These findings pave the way for understanding cell behaviour and distribution in complex geometries.

## 1. Introduction

Microorganisms play a vital role in a variety of environmental, agricultural and biological phenomena [1–3]. They are distributed widely, from deep in the ground to the digestive tract of animals. Bacteria in soil, for instance, live in heterogeneous granular matter, and their habitat is influenced by geometrical constraints, such as granular size and shape [4–8]. Understanding the behaviour of cells in such a complex geometry is thus a fundamental scientific question. This knowledge can be used to predict cell distribution [9,10], which paves the way for controlling distribution and growth in various environments. However, despite their widely recognized importance, the basic physical mechanisms that govern cell behaviour in complex geometries are still largely unknown.

The simplest geometric constraint may be a domain bounded by a flat wall. When there is a wall boundary, the cell distribution is influenced not only by biological responses but also by mechanical forces. From a fluid mechanics perspective, a cell swimming apart from a wall tends to move towards or away from the wall depending on the swimmer's type [11]. In the case of a pusher (i.e. the thrust is generated behind the body), the cell tends to swim towards the wall. In the case of a puller (i.e. the thrust is generated in front of the body), on the other hand, the cell tends to swim away from the wall. In previous experimental studies, an accumulation of pushers was observed for bull spermatozoa [12], human spermatozoa [13] and *Escherichia coli* bacteria [14,15].

In the vicinity of a flat wall, cells show various behaviours. When a unicellular freshwater ciliate *Paramecium* bumps against a solid object with its anterior end, the cell swims backwards at first, gyrates about its posterior end, and then resumes normal forward locomotion. Such a biological reaction is referred to as an avoiding reaction [16]. Ferracci *et al*. [17] used another ciliate *Tetrahymena* and showed that, due to hydrodynamic forces, it tends to swim away from a solid wall while it is trapped at a water–air interface. *E. coli* bacteria exhibited a stable circular trajectory in the vicinity of a solid wall. The entrapment mechanism has been explained by hydrodynamics as well as steric effects [18–21]. Entrapment in the vicinity of a solid wall has also been reported for spermatozoa and explained by hydrodynamic and steric effects [22–24].

**Figure 1.**
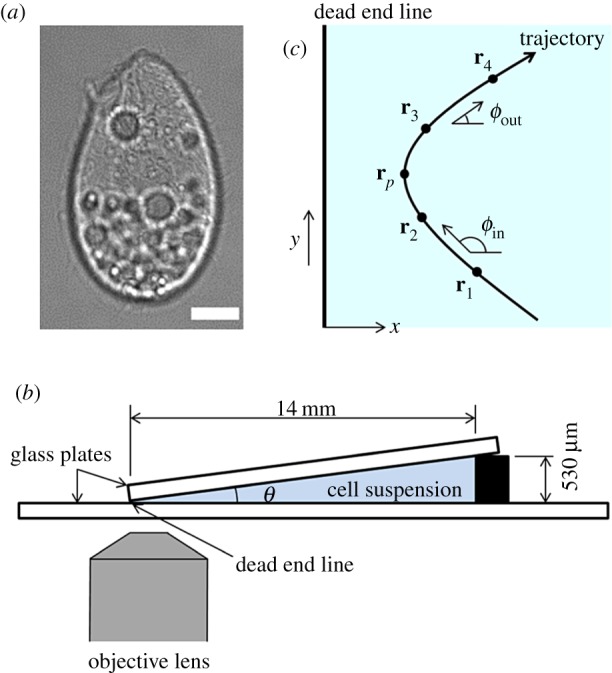
Experimental set-up and definition of parameters. (*a*) Differential interference contrast image of *T. thermophila*. Scale bar, 10 µm. (*b*) Schematic diagram of the experimental set-up. A suspension of *T. thermophila* was placed between two glass plates that met at one end and had angle *θ*. (*c*) Definition of the coordinate system and positions along a trajectory. The *x*-axis is taken perpendicular to the dead end line of two glass plates, and *x* = 0 at the dead end line. *x_p_* is the position nearest to the dead end line along the trajectory. The entry angle *ϕ*_in_ was defined as the angle between vector **r**_2_ − **r**_1_ and **x**, and the reflection angle *ϕ*_out_ was defined as the angle between vector **r**_4_ − **r**_3_ and **x**. (Online version in colour.)

Although these previous studies are helpful in understanding the behaviour of cells in the presence of a wall, an understanding of the physical mechanisms that govern cell behaviour in a complex geometry must be strengthened. Thus, as a next step, we investigated the behaviour of a ciliate between two flat plates with a small angle. Although behaviours of microswimmers in more complex geometries have been reported [25–32], we believe that the present problem setting provides a reasonable advancement regarding the behaviour of a cell near a single wall. In this study, a unicellular freshwater ciliate *T. thermophila* was used as the model microorganism (cf. figure 1*a*), because a ciliate shows both biological and physical reactions to a mechanical stimulus, which enables us to estimate the importance of physics compared with biology in describing cell behaviour near walls. Moreover, mathematical modelling of the near-field fluid mechanics of a ciliate is possible [33], and this provides insight into the behavioural mechanism from a physical point of view. We note that ciliates live in complex geometries in nature, such as in soil [34,35] and in the digestive tract [36,37].

In the following, we demonstrate that cells tend to escape from the dead end where two flat plates make contact; this occurs more by hydrodynamics than by biological reaction. In the case of hydrodynamic escape, our results showed that the cells' trajectories became almost symmetric on the way to and from the dead end. The basic characteristics of the hydrodynamic escape were well reproduced by our computational model, which illustrated that hydrodynamics assist in the cell's escape from the dead end. These findings pave the way for understanding cell behaviour and distribution in a complex geometry.

## 2. Results

### (a) Avoiding reaction and hydrodynamic escape

**Figure 2.**
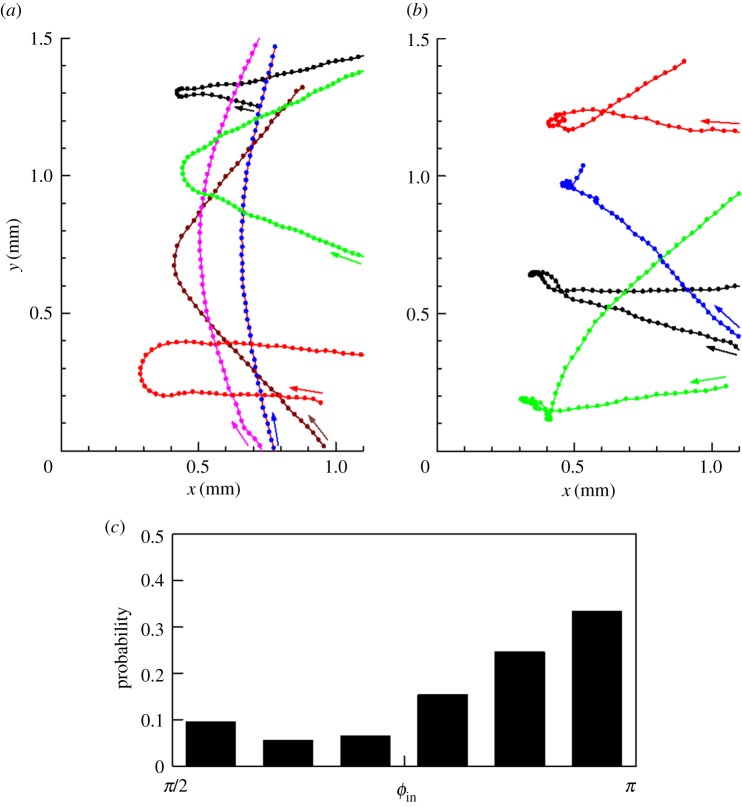
Trajectories of *T. thermophila* with or without an avoiding reaction (see electronic supplementary material, movie S1). (*a*) Six sample trajectories of hydrodynamic responses. Arrows indicate the directions of motion. Dots are plotted with 0.07 s interval. (*b*) Four sample trajectories with the avoiding reaction, in which backward swimming was observed. Dots are plotted with 0.07 s interval. (*c*) Correlation between the probability of showing an avoiding reaction and the entry angle *ϕ*_in_ (*n* = 334). (Online version in colour.)

A dilute suspension of *T. thermophila* was placed between two flat plates with a small angle, as shown in figure 1*b*. In this configuration, the geometry provided a ‘dead end' line where the two flat plates made contact. We first show trajectories of *T. thermophila* in figure 2*a,b*; *x* = 0 indicates the dead end line. A sample movie (electronic supplementary material, movie S1) is provided. As the cell approached the dead end line, the cell tended to swim away from it. Some cells showed an avoiding reaction in which cells first swam backwards, gyrated about their posterior end and then resumed normal forward locomotion. In the present study, the cell's responses involving backward swimming were classified as an avoiding reaction (cf. figure 2*b*), whereas the other responses were classified as hydrodynamic (cf. figure 2*a*). Whether the other responses were really hydrodynamic will be discussed in the following sections from the perspective of our computational model.

In the case of hydrodynamic responses (figure 2*a*), the trajectories were smooth and approximately symmetric with respect to the *x*-axis. A cell with large entry angle *ϕ*_in_ from the *x*-axis had a small reflection angle *ϕ*_out_, where the angles were defined from the *x*-axis as shown in figure 1*c*. In the case of an avoiding reaction (figure 2*b*), on the other hand, the trajectories were erratic around *x_p_*, where *x_p_* was the nearest *x* position to the dead end line. In both cases, cells tended to escape from the dead end line. Figure 2*c* shows the probability of an avoiding reaction as a function of *ϕ*_in_; the probability increased with *ϕ*_in_. The avoiding reaction appears when the cell's anterior end is strongly agitated [16]; thus, the larger *ϕ*_in_ probably induced stronger mechanical stress on the cell's anterior end. The overall probability of an avoiding reaction was about 18% in 334 trajectories, which indicates that hydrodynamic responses dominated the escaping phenomena of *T. thermophila*.

### (b) Trajectories of hydrodynamic responses

**Figure 3.**
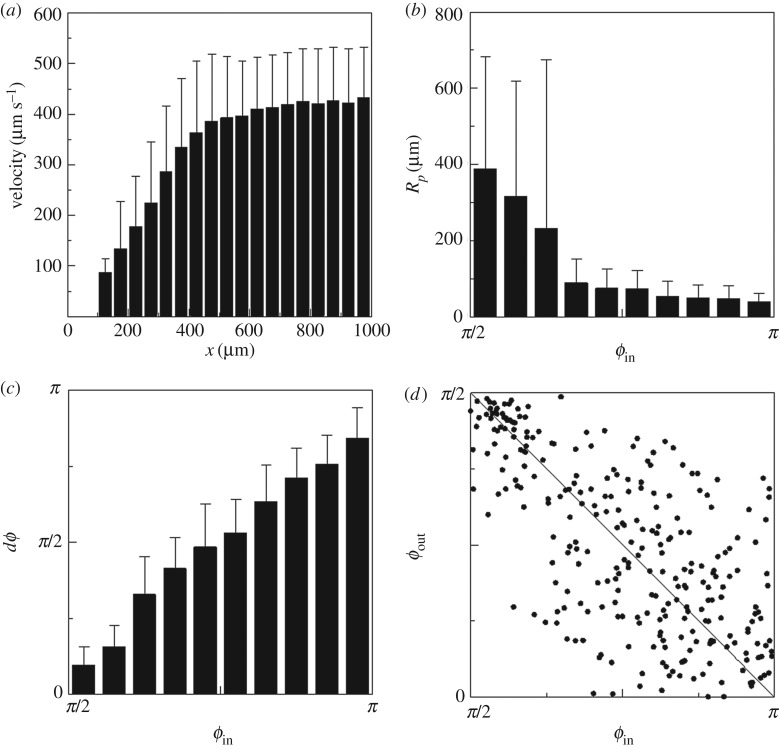
Characteristics of swimming trajectories of *T. thermophila* showing hydrodynamic responses (*n* = 274). (*a*) Correlation between the swimming velocity and the *x* position. (*b*) Correlation between the radius of curvature around *x_p_* and the entry angle *ϕ*_in_. (*c*) Correlation between *ϕ*_in_ and the angle change *dϕ* defined by *dϕ* = *ϕ*_in_ − *ϕ*_out_. (*d*) Correlation between *ϕ*_in_ and *ϕ*_out_. The correlation coefficient is −0.66, which indicates a significant negative correlation.

Next, we characterized the trajectories in the case of hydrodynamic responses. Figure 3*a* shows the swimming velocity of *T. thermophila* as a function of *x*; the velocity decreased sharply with *x*. Two possible mechanisms may be responsible for the slower swimming velocity. One mechanism is that of a lubrication force. Lubrication force increases as the clearance between the cell surface and the wall decreases. This mechanism will be validated in §2c using our computational model. The second mechanism is a disturbance in ciliary beat due to surface interactions between the cilia and wall. This mechanism will be discussed in §3.

Figure 3*b* shows the radius of curvature *R_p_* around *x_p_*. *R_p_* was large when *ϕ*_in_ was nearly π/2 (i.e. when the trajectory was nearly parallel to the dead end line). By contrast, when *ϕ*_in_ exceeded about 0.65π, *R_p_* was small. These results indicate that the rotational velocity required for the cell to escape increased with *ϕ*_in_. Such a tendency was also observed in our computational model (cf. §2c).

The trajectories shown in figure 2*a* were approximately symmetric with respect to the *x*-axis. To address the tendency quantitatively, we analysed the correlation between *ϕ*_in_ and *dϕ* (=*ϕ*_in_−*ϕ*_out_) in figure 3*c*. *dϕ* increased almost linearly with *ϕ*_in_. *dϕ* approached zero for *ϕ*_in_ = π/2; thus, a cell swimming parallel to the dead end line did not change its orientation considerably. *dϕ* was about π when *ϕ*_in_ was π, which indicates that a cell swimming perpendicular to the dead end line made a U-turn. Figure 3*d* shows the correlation between *ϕ*_in_ and *ϕ*_out_; the correlation coefficient was −0.66, which indicates a significant negative correlation between *ϕ*_in_ and *ϕ*_out_.

### (c) Trajectories obtained by simulation

**Figure 4.**
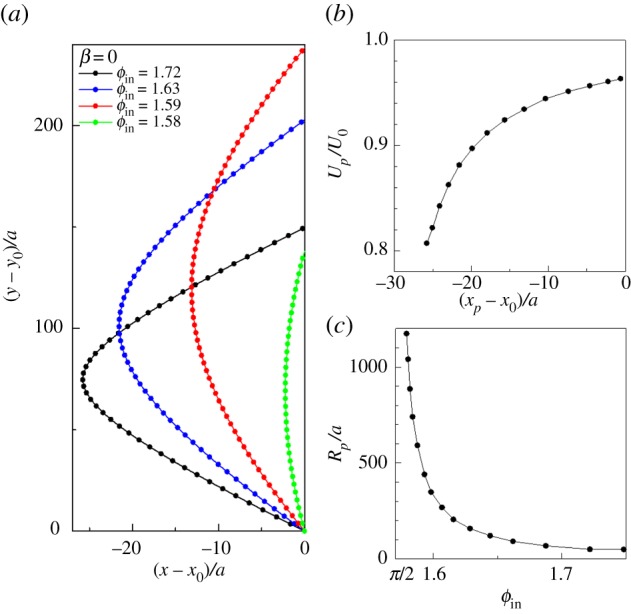
Characteristics of swimming trajectories obtained by numerical simulations (*β* = 0). *ϕ*_in_ was calculated by assuming the swimming velocity of *T. thermophila* to be 440 µm s^−1^ and the radius in *z*-direction to be 12.7 µm. (*a*) Sample trajectories of squirmers. Cells swim from the origin with different initial angle. Dots are plotted every 4*tU*/*a* time unit. (*b*) Correlation between *x_p_* and the swimming velocity at *x_p_*. (*c*) Correlation between the radius of curvature around *x_p_* and *ϕ*_in_. (Online version in colour.)

**Figure 5.**
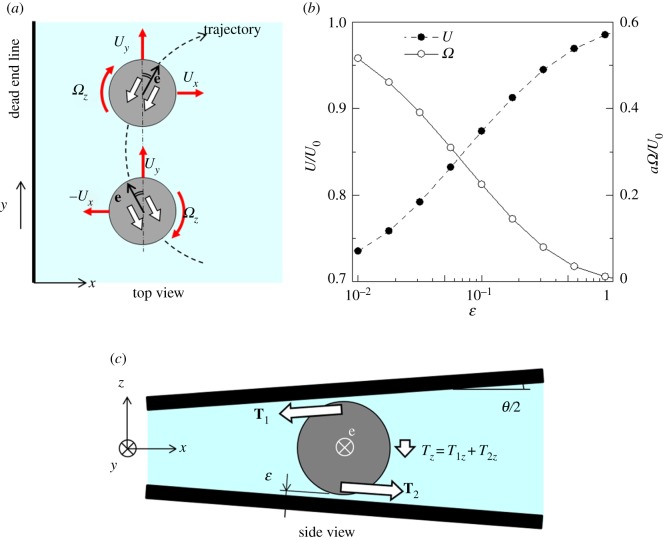
Mechanism for a squirmer with *β* = 0 to swim away from the dead end line. (*a*) Schematic diagram of translational velocity (*U_x_*, *U_y_*) and rotational velocity *Ω_z_* of a squirmer with *β* = 0 along a symmetric trajectory. White arrows indicate the squirming velocity. When the angles between **e** and the *y*-axis are opposite, the cells have same *U_y_* and *Ω_z_* but opposite *U_x_*. (*b*) Translational and rotational velocities of a squirmer near a single flat wall. The cell is directed parallel to the wall with a clearance *ɛ* from the wall. (*c*) Schematic diagram of a torque balance generated by lubrication flow, where **e** is the orientation vector of a squirmer, and **T***_i_* is a torque generated by wall *i*. (Online version in colour.)

A computer model based on fluid mechanics was constructed to determine the role of hydrodynamics in the cell movement observed in our experimental results described in §2b. The main purpose of this section is to investigate general behaviours of a ciliate using a simple mathematical model, which would lead to a better understanding of the mechanism of hydrodynamic escape in the next section. *Tetrahymena thermophila* was modelled as a spherical squirmer [38–40]. The tangential surface velocity on a squirmer was given by 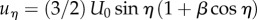, where *U*_0_ is the swimming velocity in an unbounded fluid, *η* is the polar angle from the squirmer's orientation **e**, and *β* indicates the swimming mode. The motion of a squirmer was solved by a dynamic simulation method [41,42]. The numerical results of trajectories are shown in figure 4*a*. The trajectories with *β* = 0 were symmetric with respect to the *x*-axis. The symmetry came from the governing equation of fluid mechanics with a negligible Reynolds number. In the Stokes flow regime, translational velocity (*U_x_*, *U_y_*) and rotational velocity *Ω_z_* of a squirmer with *β* = 0 can be schematically shown as figure 5*a*. When the angles between **e** and the *y*-axis are opposite, the cells have same *U_y_* and *Ω_z_* but opposite *U_x_*, which results in the symmetric trajectory.

The swimming velocity decreased as the squirmer came close to the dead end line, as shown in figure 4*b*. A slower velocity was induced by a large resistance coefficient in the grand resistance matrix (cf. §5e), which was the result of lubrication forces that increase as the clearance between the cell surface and the wall decreases.

The radius of curvature *R_p_* decreased as *ϕ*_in_ increased, which is again explained by lubrication forces. The torque to make a squirmer turn *T_z_* increased as the clearance between the cell surface and the wall decreased. The clearance decreased as *ϕ*_in_ increased; thus *R_p_* was reduced as *ϕ*_in_ increased. These results indicate that the experimental results can be qualitatively explained by hydrodynamics and that the escape of *T. thermophila* from the dead end line was strongly influenced by fluid mechanics.

### (d) Mechanism of hydrodynamic escape

To understand the mechanism of hydrodynamic escape, we performed a trial simulation of a squirmer near a single flat wall instead of two flat walls. Figure 5*b* shows the translational and rotational velocities of a squirmer near a wall when the squirmer was placed at the clearance of *ε* from the wall and with its orientation parallel to the wall. The swimming velocity *U* decreased with the clearance, which is consistent with the results shown in figure 4*b*. The rotational velocity *Ω*, on the other hand, increased as the clearance decreased, because strong lubrication torque due to surface squirming was generated in the small *ε* region. The sign of *Ω* was positive, indicating that the squirmer tended to swim away from the wall.

If two flat walls are placed in parallel, the rotational velocities generated by the two walls cancel out. Instead, two opposite torques **T**_1_ and **T**_2_ are induced from the walls, which also cancel out. When there is an angle between two flat walls, as in the present study, the torques do not cancel out completely, as shown schematically in figure 5*c*. Although the *x-* and *y-*components of torques cancel out between two walls, the *z-*component remains non-zero. The sign of *T_z_* is negative, which results in the escape rotational velocity of a cell. The mechanism of a cell's escape can be well understood by hydrodynamics.

## 3. Discussion

**Figure 6.**
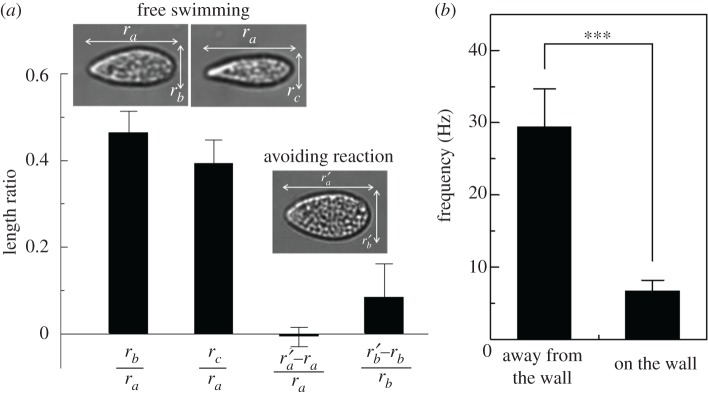
Deformation of *T. thermophila.* (*a*) Aspect ratio of cells during free swimming and that showing an avoiding reaction (*n* = 60), where *r_a_* is the body length; *r_b_* and *r_c_* are the largest and smallest body widths, respectively, while free swimming; and *r_a_′* and *r_b_′* are the body length and width, respectively, when the cell shows an avoiding reaction. The insets indicate the shapes of a sample cell during free swimming and an avoiding reaction. (*b*) Ciliary beat frequency on the wall and away from the wall, when a cell was trapped between two walls (*p* < 0.001, *n* = 50, see electronic supplementary material movie S2).

In analysing trajectories of *T. thermophila*, we realized that cells could swim in a gap narrower than their width. Because the glass wall was much more rigid than the cell bodies, cells probably deformed while swimming near the dead end line. We thus measured the deformation of each cell by following each trajectory involving both free swimming with spins and avoiding reaction near the dead end line. The results are shown in figure 6*a*. The major axis of *T. thermophila r_a_* during free swimming was about 65 µm. Two minor axes, *r_b_* and *r_c_*, were approximately 0.47*r_a_* and 0.39*r_a_*, respectively. When the cell showed an avoiding reaction near the dead end line, we measured the deformed major and minor axes, *r_a_′* and *r_b_′*, of the cell. In figure 6*a*, an increase in the minor axis *r_b_′* was evident, which indicates that the cells became deformed while swimming near the dead end line.

We next investigated how the ciliary beat was disturbed by surface interactions between the cilia and the wall. After a period of time, several *T. thermophila* became trapped and accumulated near the dead end line, probably because the cells were attracted to oxygen (i.e. chemotaxis). The ciliary beat of such a trapped cell was observed by a high-speed camera, as shown in electronic supplementary material, movie S2. Ciliary beat frequency on the wall and away from the wall were measured, and the results are shown in figure 6*b*. We see that the ciliary beat was considerably disturbed by the wall, and the beat frequency was reduced to about one-third.

**Figure 7.**
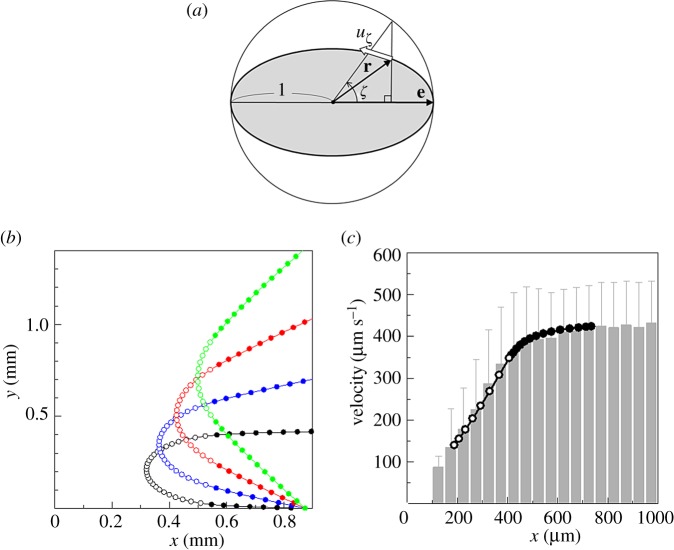
Simulation results of a deformable squirmer with *β* = 0. The results were dimensionalized using the average swimming velocity of 440 µm s^−1^ and half of the short width *r_c_*/2 = 12.7 µm. The value of *κ* was assumed to be 1.5, and the clearance at the dead end line was assumed to be 5 µm. (*a*) Definition of *ζ* and *u_ζ_*. (*b*) Trajectories of a deformable squirmer. Cells were deformed at positions indicated by open circles, whereas cells were not deformed at positions indicated by filled circles. (*c*) Comparison of swimming velocity between the experiments and the simulation. Cells were deformed at positions indicated by open circles, whereas cells were not deformed at positions indicated by filled circles. (Online version in colour.)

Although the cells were deformed and the ciliary beat significantly disturbed, the trajectories of cells were not altered dramatically by wall contact. Thus, we attempted to expand our computational model to account for cell deformation. The deformation of a squirmer was considered when the clearance between the spherical squirmer and the wall became less than 0.01*a*, as explained in §5e. We assumed that the squirmer deformed instantaneously to an oblate spheroid so as to keep the clearance of 0.01*a* and the original volume of a cell. The surface squirming velocity was assumed to be reduced by the deformation, as given by3.1
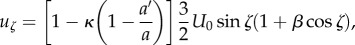
where *κ* is a coefficient for velocity reduction to account for the decrease in ciliary beat frequency (cf. Figure 6*b*), and *a′* is the half-length of the minor axis of the oblate spheroid. *ζ* is the angle defined as 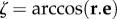 (cf. figure 7*a*), where **r** is the position on the spheroidal surface and **e** is the orientation vector.

The results of trajectories with *κ* = 1.5 and *β* = 0 are shown in figure 7*b*. The deformation of a cell was updated as the cell approached and escaped from the dead end, and the cell was deformed at positions indicated by open circles. The results were dimensionalized using the average swimming velocity of 440 µm s^−1^, half of the short width *r_c_*/2 = 12.7 µm, and the clearance at the dead end line of 5 µm. We see that, similar to the experimental results shown in figure 2*a*, the trajectories were smooth regardless of the deformation. The trajectories were again symmetric about the *x*-axis. By considering the decrease in the squirming velocity, we could express the large decrease in swimming velocity observed in the experiments (cf. figure 3*a*). Figure 7*c* shows a comparison of the swimming velocities under the experimental and simulation conditions. The simulation results were dimensionalized using an average swimming velocity of 440 µm s^−1^ and half of the short width *r_c_*/2 = 12.7 µm. By adjusting the value of *κ* to be 1.5 and the clearance at the dead end line to be 5 µm, we achieved quantitative agreement between the simulation and the experimental results. Thus, the escape of *T. thermophila* from the dead end line can be understood in terms of biomechanics, even when the cell is deformed considerably.

**Figure 8.**
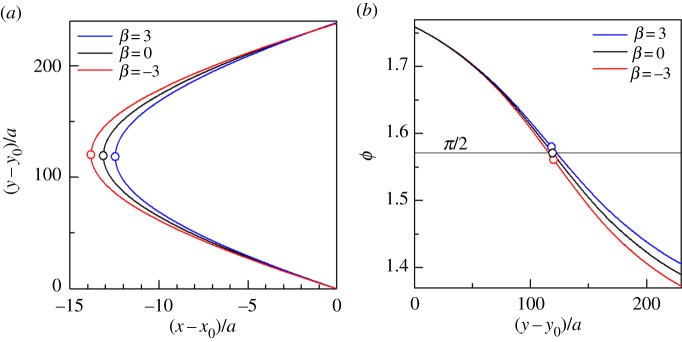
Effect of the squirming mode *β* on the trajectory of a spherical squirmer. (*a*) Sample trajectories with *β* = 3, 0 and −3. The circles indicate the positions nearest to the dead end line along the trajectories (i.e. **r***_p_*). (*b*) Change of the orientation angle *ϕ* with *y* (*β* = 3, 0 and −3). The circles indicate **r***_p_*. (Online version in colour.)

Last, we discuss how the escape phenomena will be modified by the surface squirming mode of a ciliate. The trajectories and the orientation angle from the *x*-axis *ϕ* of a spherical squirmer with *β* = ±3 are shown in figure 8. We see in figure 8*b* that *ϕ* with *β* = ±3 is asymmetric with respect to the *x*-axis, given that *ϕ* is not π/2 at **r***_p_*. Although the symmetry in a trajectory is no longer guaranteed when *β* ≠ 0, *β* had only a slight effect on the trajectory. This is because *β* does not influence the surface squirming velocity at the equator of the squirmer, where the lubrication force is maximum. The attraction force was generated between a pusher and a wall; thus, the squirmer with *β* = −3 swam closer to the dead end line compared with the neutral squirmer (*β* = 0). The repulsion force was generated between a puller and a wall, so the squirmer with *β* = 3 swam farther from the dead end line. In contrast to former studies on far-field fluid mechanics, the present escape phenomenon was not strongly influenced by the swimming mode, which clearly illustrates that near-field fluid mechanics dominates the phenomena.

## 4. Conclusion

In this study, we used *T. thermophila* as a model microorganism and experimentally investigated cell behaviour between two flat plates with a small angle. The geometry provides a dead end where the two flat plates meet. The results showed that cells tended to escape from the dead end more by hydrodynamics than by a biological reaction. In the case of hydrodynamic escape, cells' trajectories became almost symmetric on the way to and from the dead end line. When *T. thermophila* came close to the dead end line, the cells were compressed between the two flat plates while cilia kept beating with reduced frequency. These cells again showed symmetric trajectories, although the swimming velocity slowed down. These behaviours were well reproduced by our computational model based on biomechanics. The mechanism of hydrodynamic escape can be understood in terms of a torque balance induced by lubrication flow. We therefore conclude that the cell's escape from the dead end was assisted by hydrodynamics. These findings pave the way for understanding cell behaviour and distribution in complex geometries.

## 5. Material and methods

### (a) Experimental set-up

Figure 1*b* shows a schematic diagram of the experimental set-up. Cell suspension (approximately 130 µl) was first placed on a glass bottom dish (D11140H, Matsunami, Osaka, Japan). A square cover glass with a side length of 18 mm (C018181, Matsunami, Osaka, Japan) was placed over the suspension. A 530 μm-thick spacer was placed on one side, and the two glass plates were in contact on the opposite side, in which the angle between the two plates was about 2.2°. The line where the two plates made contact is hereafter referred to as the ‘dead end line'; cells could not penetrate the dead end line. The clearance at the dead end line was not precisely zero, because the top glass plate was not completely flat, and the edge of the glass plate was not completely smooth and straight. The clearance was, however, less than several microns, much smaller than the cell's body length of 65 µm.

The swimming motions of cells were observed by an inverted microscope (IX71, Olympus, Tokyo, Japan) with 4× objective lens (numerical aperture, NA: 0.75). The motion was recorded by a digital charge-coupled device (CCD) camera (DP70, Olympus, Tokyo, Japan) with a frame rate of 29 frames per second (fps); the image size was 680 × 510 pixels. The temperature was maintained at 28°C by a thermoplate (MATS-55RAF20, Tokai Hit, Shizuoka, Japan).

Cell deformation (figure 6*a*) and ciliary beat frequency (figure 6*b*) were measured using an upright microscope (BX51WI, Olympus, Tokyo, Japan) and a high-speed camera (Fastcam SA3, Photron, Tokyo, Japan). In measuring the deformation of cells, a 20× dry objective lens (NA: 0.45) was used to acquire images of size 1024 × 1024 pixels taken at a speed of 250 fps. In measuring the ciliary beat frequency, a 60 × oil immersion objective lens (NA: 1.42) was used. Images of 1024 × 1024 pixels were acquired at a speed of 1000 fps.

### (b) Cell culture

The cells used in the experiment were *T. thermophila* (wild type: CH1); these cells were cultivated and observed in PYD consisting of 1% of protease peptone, 0.5% of yeast extract and 0.87% of glucose in pure water at 28°C. The cells were cultured in a 60 mm diameter circular Petri dish (depth: 15 mm). Six to 7 days after inoculum, 20 µl of these cells were added to 8 ml of fresh medium in the Petri dish. The cells used in the experiments were 3–4 days after inoculum, when the cell growth was in the log phase. To reduce cell–cell interactions in the experiments, the number density of cells was reduced by gentle centrifugation before the experiments.

### (c) Analysis of experimental data

The trajectories of cells were obtained from recorded images using ImageJ software (NIH, Bethesda, MD, USA) with TrackMate plug-in. To remove the effect of cell–cell interactions, we neglected trajectories involving cell–cell collisions. Trajectories of cells trapped between two walls were also neglected. The obtained trajectories were analysed by in-house code. The *x-*axis was taken orthogonal with respect to the dead end line, and the *y-*axis was taken along the dead end line, as shown in figure 1*c*. We first searched the position nearest the dead end line **r***_p_* = (*x_p_*, *y_p_*) along each trajectory. Positions **r**_1_, **r**_2_, **r**_3_ and **r**_4_ in figure 1*c* were then defined as positions at 0.45 s before, 0.14 s before, 0.14 s after and 0.45 s after *x_p_*, respectively. The entry angle *ϕ*_in_ was defined as the angle between vector **r**_2_ − **r**_1_ and **x**, and the reflection angle *ϕ*_out_ was defined as the angle between vector **r**_4_ − **r**_3_ and **x**, as shown in figure 1*c*. The change in angle *dϕ* was defined as *dϕ* = *ϕ*_in_ − *ϕ*_out_. The radius of curvature around *x_p_* was calculated by drawing a circle passing through **r**_2_, **r***_p_* and **r**_3_.

### (d) Squirmer model

*Tetrahymena thermophila* was modelled as a spherical squirmer with radius *a* [38–40]. It was assumed to be neutrally buoyant and to have a very small Reynolds number during swimming. The sphere's surface was assumed to move purely tangentially, and these tangential motions were assumed to be axisymmetric and time-independent. The tangential surface velocity on a squirmer is given by 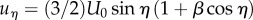, where *U*_0_ is the swimming velocity in an unbounded fluid, *η* is the polar angle from the squirmer's orientation and *β* indicates the swimming mode. A squirmer with positive *β* is a puller, whereas a squirmer with negative *β* is a pusher. The *β* value of *T. thermophila* was about zero [43]; thus, we mainly show results with *β* = 0, except for figure 8.

### (e) Numerical methods

We used a dynamic simulation method similar to that used in our former studies [41,42]. At a negligible Reynolds number, the equation of motion for a squirmer suspended in a Newtonian solvent between two flat plates can be written as 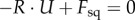, where **U** is a vector containing the translational–rotational velocities, and **R** is the grand resistance matrix constructed by superposition of pairwise interactions between an inert sphere and a wall. Pairwise additivity provides an accurate estimation of the grand resistance matrix when lubrication forces dominate the squirmer's motion, as in the present study. Exact solutions of the force–torque exerted on an inert sphere near a wall are included in standard texts [44]. **F**_sq_ is the force–torque due to the squirming motion without any translational–rotational motion, which was numerically obtained by a boundary element method [40], assuming superposition of pairwise interactions.

Initially, a squirmer was placed at (*x*_0_, *y*_0_, 0) with a clearance of 0.5*a* between the squirmer and the wall. Here, *z* = 0 indicates the centre plane between the two plates. The orientation vector of the squirmer was initially in the centre plane with a certain entry angle, and it stayed in the centre plane due to the symmetry of the problem.

In figure 7*b,c*, the deformation of a squirmer was considered when the clearance between the spherical squirmer and the wall became less than 0.01*a*. We assumed that the squirmer deformed instantaneously into an oblate spheroid so as to keep the clearance of 0.01*a*. The minor axis of the oblate spheroid was in the *z* direction, and the volume of the oblate spheroid was the same as that of the original sphere. The surface squirming velocity *u_ζ_*, which was assumed to be tangential even after the deformed shape as shown in figure 7*a*, was given by equation (3.1). *κ* was introduced because the decrease in the ciliary beat frequency was observed experimentally (cf. figure 6*b*). The force–torque exerted on an inert oblate spheroid near a wall, as well as those due to the squirming motion, were numerically calculated by a boundary element method [40].
